# Experience of tele-psychiatry during COVID-19 amongst doctors working in a mental health trust: A survey

**DOI:** 10.1192/bjo.2021.94

**Published:** 2021-06-18

**Authors:** Pallavi Chandra, Nilamadhab Kar, Abdalla Yahia

**Affiliations:** Black Country Healthcare NHS Foundation Trust

## Abstract

**Aims:**

There is paucity of information about perspectives of psychiatrists about telemedicine practice during COVID-19 pandemic. It was intended to explore the experience of doctors using tele-psychiatry for provision of clinical care during the COVID-19 pandemic in a Mental Health Trust covering four cities in West Midlands, UK.

**Method:**

The study was conducted as an anonymized questionnaire survey. A 10-item questionnaire was designed with items related to the clinical outcomes, challenges and provider satisfaction when using tele-psychiatry. It had mostly yes/no dichotomous responses along with the scope for making additional comments for each question. An online link of the questionnaire was sent via email to doctors of all grades working across the Black Country Healthcare NHS foundation Trust, in the West Midlands. The survey was open between July and October 2020; and monthly reminders were sent.

**Result:**

The questionnaire was sent out to 159 doctors and 34 responded (response rate of 21.3%). Just over 50% had used both telephone and video consultations, whereas 47.1% responders had used telephone only. More than half (55.8%) reported that the tele-psychiatry affected clinical outcomes; and it was considered positive in around half (52.9%). Most (73.5%) of the responders found that limitations or challenges of using technology impacted on delivery of care remotely. However 64.7% psychiatrists were satisfied with the process overall; and 79.4% reported that they would like to use tele-psychiatry in the future as well.

Survey captured many observations from the psychiatrists. Positive comments from the psychiatrists included expedited delivery of care, reduced non-attendance rates, as well as successful multidisciplinary meetings. Challenges in specific sub-specialties such as Child and Adolescent Psychiatry or Older Adult psychiatry were reported where complete assessments were not achieved successfully. The process was felt to be appropriate in general for stable or follow-up patients in comparison to new or acutely unwell patients. There was some worry expressed about missing out non-verbal cues which assist with mental state examination.

**Conclusion:**

Inspite of a low response rate, the survey provided some understanding about the experience of doctors practicing tele-psychiatry during pandemic. While technological challenges were acknowledged, tele-psychiatry seemed to have been accepted by a majority of doctors who are also willing to continue it in their future clinical practice. There is a need to explore in a larger sample involving both patients and clinicians about the beneficial effects of tele-psychiatry that can be incorporated in the usual psychiatric practice.

